# Air Pollution in Major Chinese Cities: Some Progress, But Much More to Do

**DOI:** 10.4236/jep.2016.713162

**Published:** 2016-12-29

**Authors:** Dorrit H. Lowsen, George A. Conway

**Affiliations:** 1 U.S. Department of State, Beijing, China; 2 Division of Global Health Protection, Center for Global Health, US Centers for Disease Control and Prevention, Atlanta, GA, USA

**Keywords:** Environmental Monitoring, Air Pollution/Air Q, Ambient Air Quality, China, PM2.5

## Abstract

**Background:**

Ambient (outdoor) air pollution has been implicated as a major cause of acute cardiovascular and pulmonary illnesses and increased risk for acute and chronic effects after chronic exposures, including mortality and morbidity. In 2008, due to persistent health concerns about its workforce and their dependents, the US Mission in China began monitoring air quality at the US Embassy in Beijing. Subsequently, monitoring stations were also established at US consulates at Shanghai (2011), Guangzhou (2011), Chengdu (2012), and Shenyang (2013).

**Objectives:**

To determine whether there have been definable trends in air quality in these five Chinese cities.

**Methods:**

Air monitoring results from each locale for accumulated PM2.5 particulate matter were calculated hourly. Accumulated data were organized, culled using a standardized set of heuristics, and analyzed for trends.

**Results:**

China’s capital city, Beijing, experienced decreased PM2.5 from 2013 through 2015, but no significant long-term downward trend from 2008 through 2015. Shanghai has not shown any definable air quality trend since 2012. Chengdu experienced some improvement in air quality since 2013, but none discernible from 2012 through 2015. Guangzhou had generally better air quality, and a downward trend since 2012. Shenyang experienced increasingly severe air pollution from 2013 through 2015.

**Conclusion:**

There appear to have been recent tangible, though modest, improvements in air quality in three large Chinese cities: Beijing, Chengdu, and Guangzhou, but no apparent progress in Shanghai, and a worrisome decline in air quality observed in Shenyang. Despite recent progress, there is a long way to go before even the cities which show improvement reach Chinese standards.

## 1. Introduction

In 2008, due to persistent health concerns about its workforce and dependents, the US Mission in China began monitoring air quality at the US Embassy in Beijing. Subsequently, monitoring stations were also established at US consulates in Shanghai (2011), Guangzhou (2011), Chengdu (2012), and Shenyang (2013). Hourly values are available online (and with a mobile app) approximately fifteen minutes after each hour of the clock. Interest in this program increased during a series of acute, severe air pollution events which occurred during persistent “thermal” inversion events (multi-day periods with trapping of air in layers) in northeastern China in 2013, the largest scale affecting many provinces and lasting from approximately January 10 through 23 (with some brief lulls), which were widely covered by the domestic and international press, and given the moniker of “airpocalypse” by many expatriates [1] [2]. China’s discussions and efforts to curtail air pollution increased markedly after this signal event, with expansion of domestic air monitoring, creation of air pollution alert thresholds and communications systems, and more widespread posting of real-time air quality observations publicly available on websites for more Chinese cities [3] [4] [5]. Sales of filtering face masks and household air cleaners increased markedly in many Chinese cities. Severe air pollution events also occurred in the Harbin city area in October of 2013 [6] [7] and Shanghai in November, 2013 [8]. A prior publication [9] analyzing diplomatic facility monitoring data from China reported marked diurnal and seasonal variations in air pollution.

## 2. Background

Ambient (outdoor) air pollution has been implicated as a major cause of acute cardiovascular and pulmonary illnesses for many years during acute air inversion events, most notoriously in the United States in Donora, PA, 1948 [10] and in London, UK in 1952 [11]. Since then evidence has accumulated for an increased risk for acute and chronic effects after chronic exposures, including mortality [12] [13] [14] and morbidity [15] [16] [17]. PM-related mortality and morbidity studies have been conducted in some severely air-polluted cities in China [18] [19]. Epidemiologic analyses of the effects of PM-related to disease morbidity have been widely conducted in hospitals, special populations and people with medical conditions [20] [21] [22].

Most worrisome among published work may be the findings of a long-term cohort study of children exposed to air pollutants in southern California, which implicates increased exposure to such pollutants in delayed lung development, an increase of new cases and exacerbations of asthma and bronchitis, and airway inflammation [23]. Estimates of negative subclinical respiratory effects, such as decreased lung function detected by spirometry, and symptoms of PM air pollution have been made in other nations [24] [25] [26] [27], as in China [28] [29] [30].

A substantial [25] [28] [29] [31] [32] [33] [34] literature describes biomedical and imputed long-term effects of air pollution on the human central nervous system from cellular to systemic effects, but with uncertainties remaining about the mechanism for such effects [32].

Over the last decade, studies have been published examining how acutely the onset of symptoms and/or deleterious physiologic/pathologic changes may occur after exposure to air pollutants. For example, a double-blinded, randomized crossover study found that “Brief exposure to dilute diesel exhaust promotes myocardial ischemia and inhibits endogenous fibrinolytic capacity in men with stable coronary disease… within 15 minutes from the start of exposure to dilute diesel exhaust.” [35]. While this study alone (and un-replicated) might not fully implicate such exposure, it is consistent with other published evidence for exposure to air pollution worsening symptoms of angina pectoris in a multi-center study [36] and exacerbating myocardial ischemia during exercise [37] [38].

Fine, or PM2.5 particles, markedly smaller than PM 10, can travel into the end airways and alveoli, and many of the components may directly enter the bloodstream from there. Thus, the effects of PM 2.5 may be more insidious than larger particles, with marked systemic effects. Recent studies demonstrate rapid electrocardiographic, physiologic and symptomatic changes after exposure to PM2.5 particles and related aerosols such as diesel fumes. “These effects do not appear to be present immediately following the exposure but can be demonstrated from as early as 2 h after the exposure….” [39]. The well-documented relationship between PM2.5 and health effects, as well as the synthesis of many hazardous air quality fractions in this single measure, is why many air quality monitoring systems collect real-time PM2.5 as a sole or key indicator of air quality, and why we focus this study on that [aggregate] measure [40].

Thus, sudden changes in the severity of air pollution may have rapid effects on persons with asthma and appear to have very specific, quantifiable effects on persons with existing coronary disease, particularly those with ischemia and a history of angina pectoris. This, in turn, would support the need for as frequent and accurate measurements of air quality as possible, and the sharing of such information on a real-time basis. This information may be useful in providing warnings to susceptible individuals when conditions deteriorate, and reassurance that it is now safer to go outside to exercise when those conditions improve.

## 3. Objective

To determine whether there have been definable trends in air quality in these five Chinese cities.

## 4. Methods

Air monitoring is conducted around the clock in each locale using one or two Met One Instruments Beta Attenuation monitors (BAM 1020). Monitor placement was determined in consultation with experts from the US Environmental Protection Agency (EPA) and varies by location, but is generally consistent with air mass placement with good exposure from all sides as defined in Table 7-1 of the EPA’s Quality Assurance Handbook for Air Pollution Measurement Systems [41]. Monitors are serviced and calibrated monthly with additional servicing provided as needed in the event of unexplained downtime or anomalous readings. The monitors take hourly measurement of accumulated PM2.5 particulate matter (which can include carbon [in elemental and organic forms], sulfate [SO_2_] and nitrate [NO_x_] [often dissolved in water droplets, particularly during foggy and static air conditions], ammonium, silicon, and sodium ion, as well crustal matter and trace elements, including heavy metals) [42] [43] [44] used to calculate an average concentration in µg/m^3^ for the hour, consistent with EPA standards for measurement schedule [41]. These measurements are converted using a standardized algorithm to a stepwise-linear scale, the Air Quality Index (AQI) using the formula AQI = [(AQI_H_ − AQI_L_)/(BP_H_ − BP_L_)](Con − BP_L_) + AQI_L_ where Con represents the measured concentration, BP_H_ and BP_L_ represent the top and bottom values for the breakpoint range from Table 1 in which Con falls, and AQI_H_ and AQI_L_ represent the top and bottom of the corresponding AQI range [45]. The resulting AQIs, along with the (underlying) PM2.5 concentrations are posted to US Mission China website (URL: http://stateair.net) and Twitter feeds (Handles: @BeijingAir, @CGShanghaiAir, @Guangzhou_air, @CGChengduAir) along with health/activity warnings as indicated.

The raw data presented here are all available for download at the US Mission China air quality website, http://stateair.net.

The data were organized using a simple Microsoft Excel spreadsheet (various versions were used over the course of the work) with Excel’s built in functionality used to provide the following clean up and statistical analysis.

First, data were cleaned to close gaps. Single hour gaps in the raw data were filled using the arithmetic mean of the two surrounding data points. Multi-hour gaps in the raw data were not filled. The hourly data were then used to calculate daily, monthly, quarterly, half yearly, and annual averages (arithmetic mean), excluding from analysis any periods of time with too many missing data points resulting from multi-hour gaps. Time periods were excluded if data were available for fewer than half of the expected single-hour readings. The expected number of single-hour readings were calculated as 24 * approximate number of days in the period (year = 365, half year = 182, quarter = 91, month = 30, day = 1). Finally, trends were calculated across comparable time periods from year to year (e.g. first quarter of each year in the data set) using the linear regression tool from Excel’s Data Analysis toolkit which utilizes LINEST function to fit a line using the least squares method [46] [47].

In addition to examining trends, various intervals were evaluated for attainment of Chinese national air quality standards [48], as well as how often each city would meet international guidelines set by the World Health Organization [49]. Given the purpose of the US Mission China’s monitoring program, the percentage of days which had mean values falling below US air quality standards [50] were also calculated.

## 5. Results

**Figure 1:**
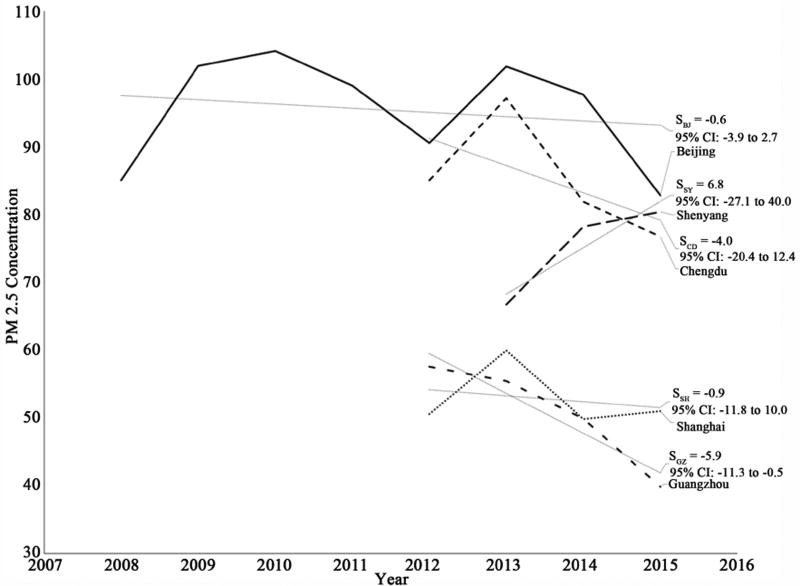
Annual average PM2.5 concentrations, 2008–2015.

**Figure 2:**
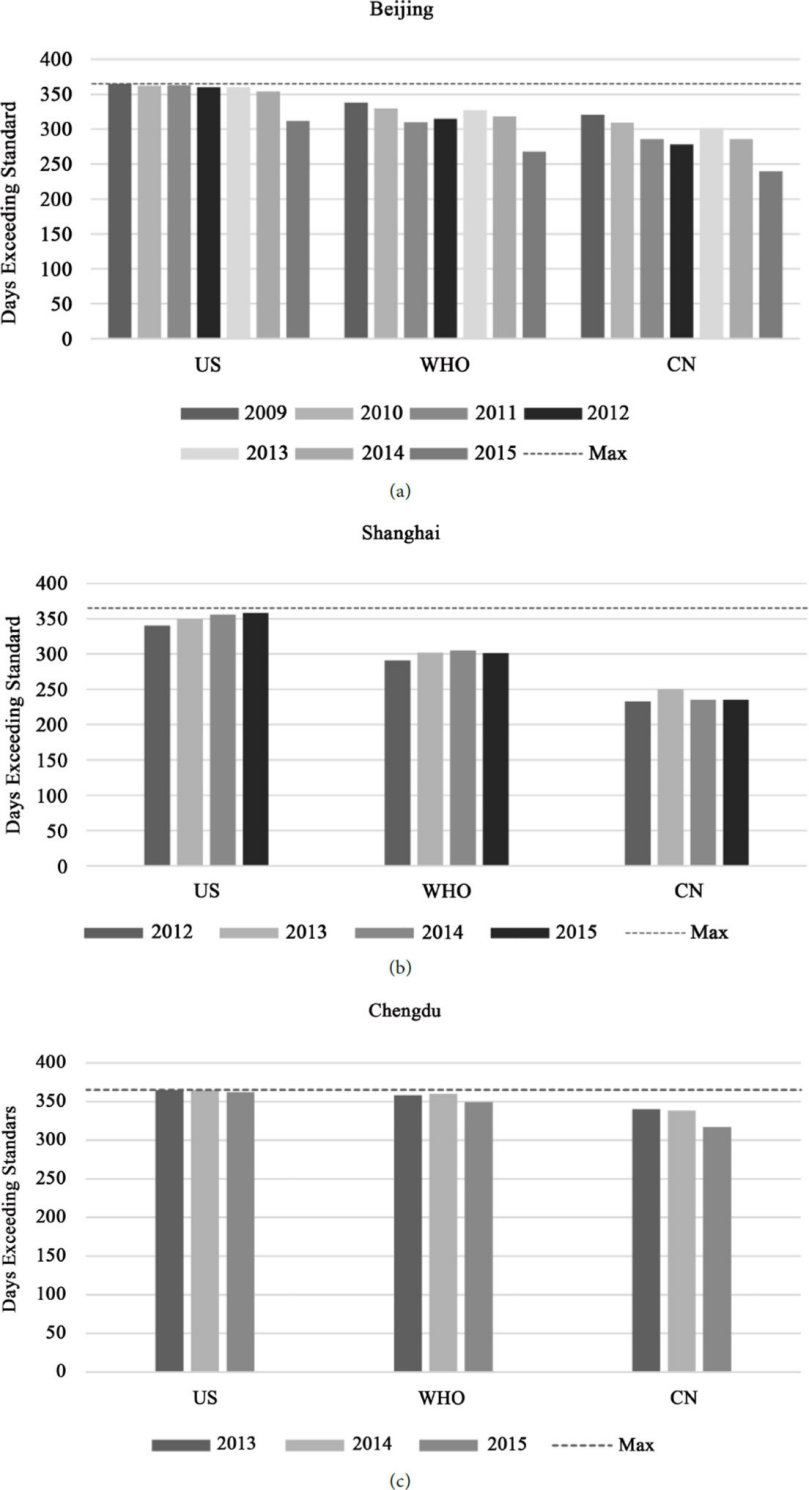

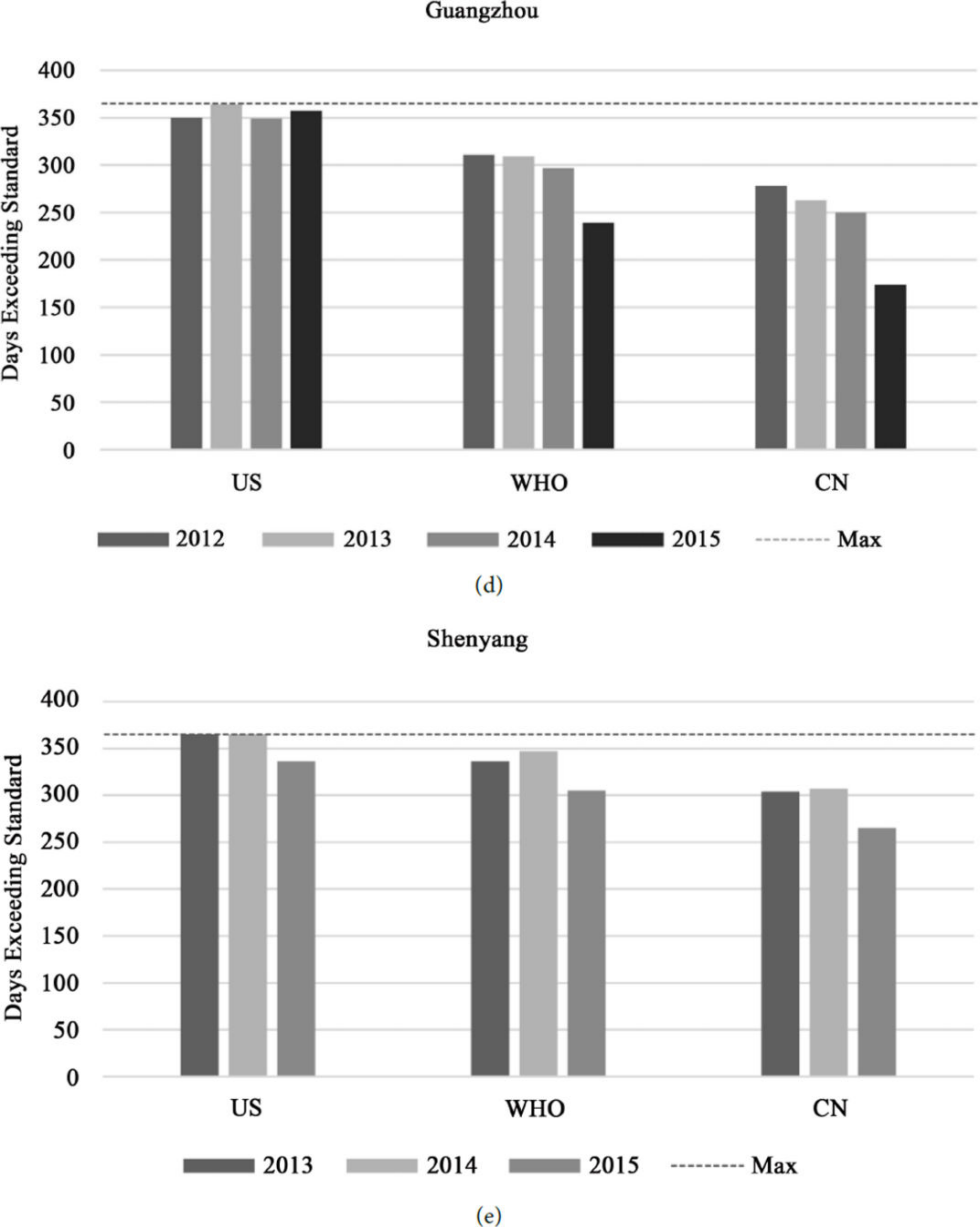
Number of days each year exceeding current US and Chinese air quality standards and WHO guidelines.

China’s capital city, Beijing, experienced a decrease in PM2.5 from 102 µg/m^3^ in 2013 to 83 µg/m^3^ in 2015, the lowest annual average since U.S. Embassy monitoring began in 2008. However, despite these apparent improvements in recent years, the data do not show a statistically significant downward trend since the beginning of the monitoring program (Figure 1 and Table 2). For 2015, 28.5 % of Beijing’s days met Chinese air quality standards, 20.1 % of days met WHO guidelines, and 6.9 % of days met US air quality standards (Figure 2 and Table 3).

China’s most populous city, Shanghai, while it does not usually suffer as severe air pollution on average as Beijing, has not shown any clear trend since monitoring began in 2012 (Figure 1 and Table 2), nor improvement in attainment of Chinese air quality standards over that interval (Figure 2 and Table 3).

Chengdu, the westernmost large city in China and capital of Szechuan Province, has experienced an apparent improvement in air quality since 2013, but not since 2012 (Figure 1 and Table 2). The number of days meeting air quality standards remains low (Figure 2 and Table 3).

Guangzhou, the southernmost city of China with a US consulate, has generally better air quality than the other cities monitored, with a downward trend since 2012 (Figure 1 and Table 2), but still exceeded Chinese air quality standards 47% of days in 2015, and seldom fell below US standards (Figure 2 and Table 3).

Shenyang, in northeastern China, while generally having somewhat better air quality throughout this interval than much-larger Beijing, experienced increasingly severe air pollution from 2013 through 2015, with its mean PM2.5 concentrations converging on those of Beijing (Figure 1 and Table 2) by 2015.

## 6. Discussion

A number of factors may affect the conclusions drawn from the data reported in this study. First, each US Mission China facility measures air quality in its immediate vicinity, so may not be fully representative of its city as a whole. In fact, in November of 2015 the Beijing Environmental Protection Bureau announced that Beijing’s air quality in the first ten months of 2015 had improved 21.8% over the same period in 2014 [51], while the U.S. Embassy’s data showed a more dramatic improvement of 28.9%.

It is outside the scope of this study to evaluate causes of improvement (or decline) in air pollution although it is worth noting for future research some factors which should be considered. It is generally accepted that meteorological conditions including wind, temperature, and humidity can affect local air pollution and its measurement [4] [28]. With the right data and analysis, it might be possible to evaluate whether the recent improvement trend observed, particularly in Beijing in 2014 in preparation for the Asia-Pacific Economic Conference and 2015 before the “70^th^ Anniversary of the Victory of the Chinese People’s War of Resistance Against Japanese Aggression and the World Anti-Fascist War” [52], directly reflects improvements resulting from successful limitation of pollution inputs (e.g., coal combustion for power and heat generation reductions and/or smokestack pollution controls, diesel exhaust from heavy transport vehicles, automotive exhausts, construction dusts, etc.) or from other factors, such as air movement (wind) and the pace of industrial production and consumption due to economic shifts. Such analysis would help us to more effectively discern whether recent rates of improvement are likely to be sustainable or are primarily the result of either factors outside human control (e.g. weather) or one-time changes (e.g. Beijing’s recent closure of most coal-fired power plants within the city’s core) which represent real gains, but not on-going year-over-year improvements. If recent rates of improvement in Beijing, Chengdu, and Guangzhou prove to be sustainable, those cities could reach Chinese, and even international air quality standards [48] within just a few years. If, on the other hand, recent improvements prove to have resulted from weather conditions, or as these authors think possible, from specific but not repeatable control measures such as closure of coal-fired power plants, or ones which may strain available public acceptance and transportation infrastructure, e.g., implementation of alternate-day driving restrictions, then the rate of improvement would likely level off, leaving attainment of air quality standards unpredictably far in the future.

It should also be noted that PM2.5 measurements may not capture the full hazards for air pollution, which are also posed by ozone, carbon monoxide, volatile organic compounds, ultrafine particles and possibly as-yet-unknown fractions.

A fractionated analysis of the PM2.5 measurements described here would provide a more granular picture, and might more precisely reflect relative hazards and trends, but are beyond the scope of this study. Interestingly, a published study of such fractions in Beijing during the highly polluted winter of 2012–2013 demonstrated rapid conversion of gas phase to particle phase for nitrates and (especially) sulfates [42].

Most importantly, this study only measured the severity of air pollution through time. The arduous process of gathering a wide range of health data and examining their frequency and temporal relationship to worsening and/or improvements in air pollution should continue apace.

## 7. Conclusion

These data provide longitudinal detail for air quality in multiple Chinese cities. There do appear to have been tangible, though modest, recent improvements in air quality in three large Chinese cities, Beijing, Chengdu, and Guangzhou, but no apparent progress in Shanghai, and a worrisome decline in air quality observed in Shenyang. Despite recent progress, there is a long way to go before even the cities which show improvement reach Chinese standards (let alone the more rigorous goals set by the WHO guidelines and US standards). If recently-observed rates of improvement persist, that could happen quickly, but our data are insufficient to conclude that those rates are durable. Relying on the longer history from Beijing, which shows little or no overall improvement since 2008, despite recent gains over peak pollution levels, the data suggest it could be a very long time before China reaches safe air quality. This could be interpreted as putting many adults at increased risk of cardiopulmonary and other diseases, and consigning a generation of Chinese children in those locales to diminished pulmonary capacities and other hazards.

## Figures and Tables

**Table 1:** Air quality index breakpoints [45].

Category	Concentration Breakpoint (BP)		Air Quality Index (AQI)	

	Low	High	Low	High
**Good**	0	12	0	50
**Moderate**	12.1	35.4	51	100
**Unhealthy for Sensitive Groups**	35.5	55.4	101	150
**Unhealthy**	55.5	150.4	151	200
**Very Unhealthy**	150.5	250.4	201	300
**Hazardous**	250.5	500.4	301	500

**Table 2:** Annual average PM2.5 concentrations 2008–2015, Chinese cities with US Mission monitoring.

Year	Beijing	Shanghai	Chengdu	Guangzhou	Shenyang
**2008**	85				
**2009**	102				
**2010**	104				
**2011**	99				
**2012**	91	51	85	58	
**2013**	102	60	97	55	67
**2014**	98	50	82	50	78
**2015**	83	51	77	40	80

**Table 3:** Percentage of days each year meeting current US and Chinese air quality standards and WHO guidelines.

**China**	**2008**	**2009**	**2010**	**2011**	**2012**	**2013**	**2014**	**2015**	**Mean**

**Beijing**	18%	15%	17%	23%	25%	17%	22%	29%	21%
**Shanghai**					37%	32%	36%	37%	35%
**Chengdu**					6%	8%	8%	13%	9%
**Guangzhou**				14%	32%	29%	33%	53%	37%
**Shenyang**						27%	16%	22%	21%

**WHO**	**2008**	**2009**	**2010**	**2011**	**2012**	**2013**	**2014**	**2015**	**Mean**

**Beijing**	9%	10%	10%	16%	15%	10%	13%	20%	13%
**Shanghai**					21%	17%	17%	18%	18%
**Chengdu**					2%	2%	1%	4%	3%
**Guangzhou**				6%	20%	16%	20%	35%	22%
**Shenyang**						13%	5%	10%	9%

**US**	**2008**	**2009**	**2010**	**2011**	**2012**	**2013**	**2014**	**2015**	**Mean**

**Beijing**	2%	0%	1%	1%	2%	1%	3%	7%	2%
**Shanghai**					7%	4%	2%	2%	4%
**Chengdu**					2%	0%	0%	1%	0%
**Guangzhou**				0%	6%	0%	5%	2%	3%
**Shenyang**						0%	0%	1%	0%
